# Results from e-KISS: electronic-KIOSK Intervention for Safer Sex: A pilot randomized controlled trial of an interactive computer-based intervention for sexual health in adolescents and young adults

**DOI:** 10.1371/journal.pone.0209064

**Published:** 2019-01-23

**Authors:** Taraneh Shafii, Samantha K. Benson, Diane M. Morrison, James P. Hughes, Matthew R. Golden, King K. Holmes

**Affiliations:** 1 Department of Pediatrics, Division of Adolescent Medicine, University of Washington School of Medicine, Seattle, WA, United States of America; 2 Harborview Medical Center, University of Washington School of Medicine, Seattle, WA, United States of America; 3 School of Social Work, University of Washington, Seattle, WA, United States of America; 4 Department of Biostatistics, University of Washington, Seattle, WA, United States of America; 5 Department of Medicine, University of Washington School of Medicine, Public Health Seattle & King County HIV/STD Program, Seattle, WA, United States of America; 6 Departments of Global Health and Medicine, University of Washington Schools of Medicine and Public Health; and Center for AIDS & STD, Seattle, WA, United States of America; University of Pennsylvania, UNITED STATES

## Abstract

**Introduction:**

Interactive computer-based interventions (ICBI) are potentially scalable tools for use in real-world settings to promote sexual health and prevent sexually transmitted infections (STIs) and unintended pregnancies. We developed and assessed the feasibility and acceptability of an ICBI for promoting adolescent and young adult sexual health, and the effectiveness of the intervention in reducing unprotected sex, STIs, and unintended pregnancy.

**Methods:**

This pilot randomized controlled trial enrolled STI Clinic patients, in Seattle, Washington, who were 14–24 years old and reported unprotected vaginal sex during the last 2 months. Both the control and intervention group used a computerized survey to enter their sexual health and only the intervention group received the ICBI. The ICBI included personalized sexual health feedback from a physician avatar; instructive video modules advocating sexual health; and identification of one behavior to change. At 3-month follow-up, participants reported on interim sexual and pregnancy histories and underwent repeat STI testing. We assessed intervention impact on unprotected vaginal sex, number of sexual partners, incident STIs, and unintended pregnancy.

**Results:**

Of 272 participants, 242 (89%) completed the study, of whom 65% were female. While these findings did not reach statistical significance, at 3-month follow-up, the intervention group reported a 33% lower rate of unprotected vaginal sex (no condom use) [IRR = 0.67, 95% CI: 0.44–1.02]; 29% fewer sex partners [IRR = 0.71, 95% CI: 0.50–1.03]; and 48% fewer STIs [IRR = 0.52, 95% CI: 0.25–1.08] when compared to the control group. Similarly, as compared to the control group, intervention females reported a lower rate of unprotected vaginal sex (no birth control) [IRR = 0.80, 95% CI: 0.47–1.35] and half as many unintended pregnancies (n = 5) versus control females (n = 10) [IRR = 0.51, 95% CI: 0.17–1.58]. In exploratory analyses, intervention females reported fewer partners [IRR = 0.71, 95% CI: 0.50–1.00] and a significantly lower rate of vaginal sex without condoms [IRR = 0.50, 95% CI: 0.30–0.85].

**Conclusion:**

The intervention was acceptable to both males and females, and at 3-month follow-up, there were non-significant reductions in risk behavior for all outcomes. Among females, exploratory analysis showed a significant reduction in vaginal sex without condoms.

## Introduction

Rates of sexually transmitted infections (STIs), including human immunodeficiency virus (HIV) infection, in adolescents and young adults ages 15 to 24 years remain at epidemic levels in the United States [1–4] accounting for half of all new cases of STI and one fifth of all new cases of HIV diagnosed per year [4, 5]. While pregnancy rates in adolescents and young adults have decreased in recent years, rates in the United States remain the highest among developed countries [6–8]. Unintended pregnancy is associated with induced abortion and pregnancy during adolescence is associated with negative outcomes for both mother and infant [9].

Researchers, clinicians, teachers, and parents continue to search for the most effective ways to discuss sexual health with adolescents and young adults to promote healthy sexual relationships and reduce risky sexual behaviors that lead to STI/HIV infection and unintended pregnancy. Behavioral interventions, such as client-centered counseling or health education curricula delivered by trained providers, have shown promise in reducing risky sexual behaviors in some populations [10–17] but are fraught with the disadvantages of variable delivery dependent on the training and expertise of the provider; prohibitive expense to establish and maintain outside of research programs; and impracticality of implementing broadly. The extensive training of healthcare providers, or the hiring and training of health educators to deliver such interventions [11, 14–19], is not feasible for many sites that serve adolescents and young adults. In addition, intervention content delivery may be time-intensive and require multiple sessions and return visits, creating logistical barriers for the intended populations to receive the complete intervention [14, 15, 19].

Computer-based interventions (CBI) have several advantages over face-to-face counseling-based interventions: (1) the prescribed intervention is delivered with fidelity; (2) there are static costs for hardware and software; and (3) the computerized format may be easily up-scaled or revised. There is evidence that computer-based interventions for health behavior change may be more effective than interventions delivered face-to-face [20, 21]. Interactive computer-based interventions (ICBI) require users to engage with the computer by entering information prompting feedback. ICBIs are emerging as effective strategies to target many health issues in adolescents and young adults including cigarette and marijuana smoking; violence [22]; alcohol abuse [23, 24]; and sexual health [12, 25, 26].

Despite the potential scalability of sexual health ICBIs, they have yet to be implemented broadly, and most have not been tested in real-world settings with real-world users. Of the studies identified in the literature [12, 27–39] only three were conducted in clinic settings [27, 28, 40, 41]; most interventions required multiple sessions over multiple visits; and the majority targeted condom use and STI/HIV prevention *or* birth control use and pregnancy prevention. Only two studies targeted dual use outcomes [13, 27, 28] and only two included biomarker outcomes [28, 40].

There is a need for brief interventions that appeal to adolescents and young adults, are practical and easily adopted into real-world settings, and assess effectiveness demonstrated by sexual behavioral and biomarker outcomes. This pilot randomized controlled trial tests the acceptability and feasibility of an ICBI for sexual health in adolescents and young adults, delivered as one brief intervention session in an STI Clinic. The objectives of this study were to: 1) demonstrate the feasibility and acceptability of an interactive computer based intervention for sexual health; 2) assess the effectiveness of the intervention in reducing unprotected sex between groups at 3 months; and 3) pilot test the biomarker outcomes of *Chlamydia trachomatis* (CT) and *Neisseria gonorrhoeae* (GC) infections and pregnancy.

## Materials and methods

A research assistant recruited males and females, 14–24 years of age, from the waiting room of a public health STI Clinic in Seattle, Washington. They were screened for eligibility and gave assent or consent via computer.

With approval of the University of Washington (UW) Human Subjects Division, prior to participation in the study, assent was obtained from those participants less than 18 years old and consent was obtained for those 18 years and older. As approved by the UW Human Subjects Division, parental consent was waived for participants under 18 years of age, as 14 years is the legal minimum age to consent for STI/HIV services in Washington State. A partial waiver of consent was also granted by UW Human Subjects Division to omit information about randomization into the control or experimental group to receive an ICBI for safer-sex as we were concerned this knowledge may influence participant answers and bias the study results.

To enhance recruitment, fliers advertising the study were posted at locations in the community targeting demographically similar populations. These locations included two medical clinics and one drop-in center serving homeless youth; one pediatric clinic and one family practice clinic, both providing care to underserved populations. A research assistant screened interested participants from these locations for eligibility by telephone.

To be eligible for the study participants were required to speak and read English; report at least one episode of unprotected (either no condom or no birth control: e.g. pills, patch, injection, ring, intrauterine device (IUD) and implant) vaginal sex in the last two months; report no current pregnancy in self or vaginal sex partners; and not actively seeking pregnancy. In addition, for follow-up and retention, participants were asked to provide contact information for self and friends or family. Females were screened by urine pregnancy test and excluded if pregnant. Pregnant females were counseled by a clinician and offered referral to obstetric medical care.

At the baseline visit all participants entered their demographic information and sexual history via computer assisted self-interview (CASI) and provided urine samples for STI testing. There were two study computers and one research assistant and on the rare occasion when there were two participants in clinic at the same time each completed the study in a private clinic room and had a different study start time. They did not know how long the other participant had been on the computer as they were in different rooms and once each participant finished the study they continued with their clinic visit. After entering their sexual history into the computer, the control group continued their clinic visit per the clinic’s standard of care. Each intervention participant then received an interactive computer-based intervention before continuing with the routine clinic visit. If evaluated by a clinician during the visit (per clinic standard some patients undergo STI testing without seeing a clinician), participants in both allocation arms completed a short exit interview with the research assistant to identify what safer-sex topics they discussed with the clinician during the visit.

The intervention is based on concepts adopted from the Options Projects developed by Jeffrey Fisher [42]. The Options Project is a brief clinician-delivered intervention with elements of motivational interviewing, and uses the theory-based model of Information, Motivation and Behavioral Skills [43]. The Options Project was initially tested in a population of substance-addicted, HIV-positive adults [44] and delivered over the course of multiple and frequent provider visits. The intervention in the present study was designed for a one-time clinic visit with healthy adolescents and young adults. Instead of being delivered by a clinician, the intervention was presented by a computer to mimic a clinician-patient risk reduction interaction. A Seattle technology company, DatStat Inc., assisted in the computer programming of the intervention. The intervention was pilot tested with five participants to assess usability and comprehension.

Participants in the intervention group were provided with three options for interacting with the computer: using a static male physician avatar, a female physician avatar (photographic images of a provider conversing as if with the patient) or no avatar (text only). They then received personalized feedback about their protective and risky sexual behaviors from the avatar or via text only.

The computer program identifies all risky and protective behaviors as reported in the participant’s sexual history and the avatar presents the results to them. For example, in a screen shot the avatar states, “I see you are protecting yourself by 1) using birth control every time you have sex and 2) talking with your partners about using condoms.” In the next screen shot the avatar states, “But I am worried that you are at risk for STI/HIV because 1) You don’t use condoms every time you have sex and 2) You and your partners don’t get tested for STI/HIV before you have sex for the first time.”

After the feedback was provided participants were asked to choose what they want to discuss further: STI/HIV and male condom use, or birth control use and unintended pregnancy. Participants were asked to rank, on a scale from 1 to 10, the perceived importance of using condoms or birth control and how confident they were that they could use condoms or birth control more consistently. Depending on each participant’s relative importance and confidence rankings, the avatar asked why using condoms or birth control was important to the participant; what were the participant’s perceived barriers to using condoms or birth control; and what information and skills were needed to increase use of condoms or birth control. Participants were offered video modules targeting sexual health knowledge and skills, including demonstrations of how to use condoms and birth control (e.g. pills, patch, injection, ring, IUD, and implant) [45]; a vignette of a teenage couple dispelling pregnancy prevention myths [46]; and a vignette of a young adult couple negotiating condom use with each other [47]. At the end of the intervention, each participant was asked to identify a sexual risk behavior they planned to change, and to make it a personal goal to adopt that change before the study follow-up visit in 3 months.

At the three-month follow-up visit, intervention and control group participants completed the follow-up survey. They entered their interim sexual histories electronically via CASI. These questions were similar to those at the baseline survey except they accounted for the follow-up time period. Topics included condom and birth control use with vaginal intercourse; number of sex partners; and interim diagnosis of STI or pregnancy since baseline interview. Each participant in the intervention group was asked additional questions about what progress had been made toward their behavior change goal. Participants in both groups provided urine samples for STI testing and females provided urine for pregnancy testing. Participants each received USD 25 for completing the baseline visit and USD50 for completing the 3-month follow-up visit. They were also provided bus tickets home from each visit if needed.

The primary outcome at follow-up was the number of unprotected (no condoms) sex events during the last 2 months. Secondary outcomes included the number of unprotected (no birth control as reported by females) sex events during the last 2 months; the number of sex partners during the last 2 months; and incident CT and GC infection and unintended pregnancy. Aptima test kits for CT and GC were provided by GenProbe.

Sample size was calculated for the outcome of unprotected (no condom) sex in the last 2 months. The study had 80% power to determine a risk difference of 14 percentage points in the rate of unprotected sex in the last 2 months between the two treatment arms with a 2-tailed test and alpha value of 0.05. A sample size of 460 was chosen to allow for an anticipated 16% loss to follow-up, yielding outcome data on 392 participants. Due to slow recruitment, our final sample was 272. NQuery Advisor by StatsSols version 3.0 was used to calculate sample size. STATA 12.0 by StataCorp LLC was the statistical software used in the analysis. The level of significant was defined at p<0.05.

Randomization was computer generated and research staff and participants were blinded to the allocation to intervention or control arm. As each participant gave consent and was enrolled into the study via computer, allocation to treatment arm and subsequent delivery of the intervention all occurred in one sitting using the same computer while the participant was alone in a private clinic room. Research staff were blinded to group assignment for the duration of the participant encounter. Participants were also blinded as to whether or not they were receiving the intervention. During the consent process participants were informed that researchers were testing a new method to discuss sexual health with adolescents and young adults.

Randomization was stratified by gender (male or female); age (14–18 years or 19–24 years); and clinic visit type (expedited visit or clinician visit). These group determinations for stratification were based on psychosocial developmental and life-stage differences between adolescents and young adults. As standard of care in the STI Clinic at that time, all patients were screened for risk behaviors at intake and triaged to the needed level of care, either to a clinician visit with a mid-level provider or to an expedited visit where patients provide specimens only for testing without a clinician encounter. Visit type was included in the stratification to account for different levels of care that participants may have received during their visit and, therefore, varying levels of exposure to risk-reduction discussions with a clinician.

Approval for this study was granted on June 16, 2011 by the Human Subjects Division of the University of Washington. Recruitment began February 2, 2012 and all follow-up completed by June 2013. This is a pilot study and the funding agency did not consider it a randomized clinical trial, therefore it was not registered. After study completion and in consultation with staff at clinicaltrials.gov, as a conservative measure, we registered the study as a clinical trial. The authors confirm that all ongoing and related trial for this intervention are registered. This study is registered with ClinicalTrials.gov identification number: NCT03027531 https://clinicaltrials.gov/show/NCT03027531.

### Statistical methods

T-test and chi square were used to assess differences between allocation arms at baseline in demographic characteristics and reported sexual behaviors. The primary outcome of unprotected sex (no condom use), and the secondary outcomes of number of sex partners and unprotected sex (no birth control as reported by females), were compared between the intervention and control groups at 3 months. Poisson regression with robust error variance was used to account for skewed responses to count variables. Binomial regression was used to model the secondary outcomes of incident CT and GC infection and unintended pregnancy (females only). Models were also adjusted for baseline differences between intervention and control group for self-reported history of STI; ever transactional sex; and birth control use (females only).

## Results

**Fig 1:**
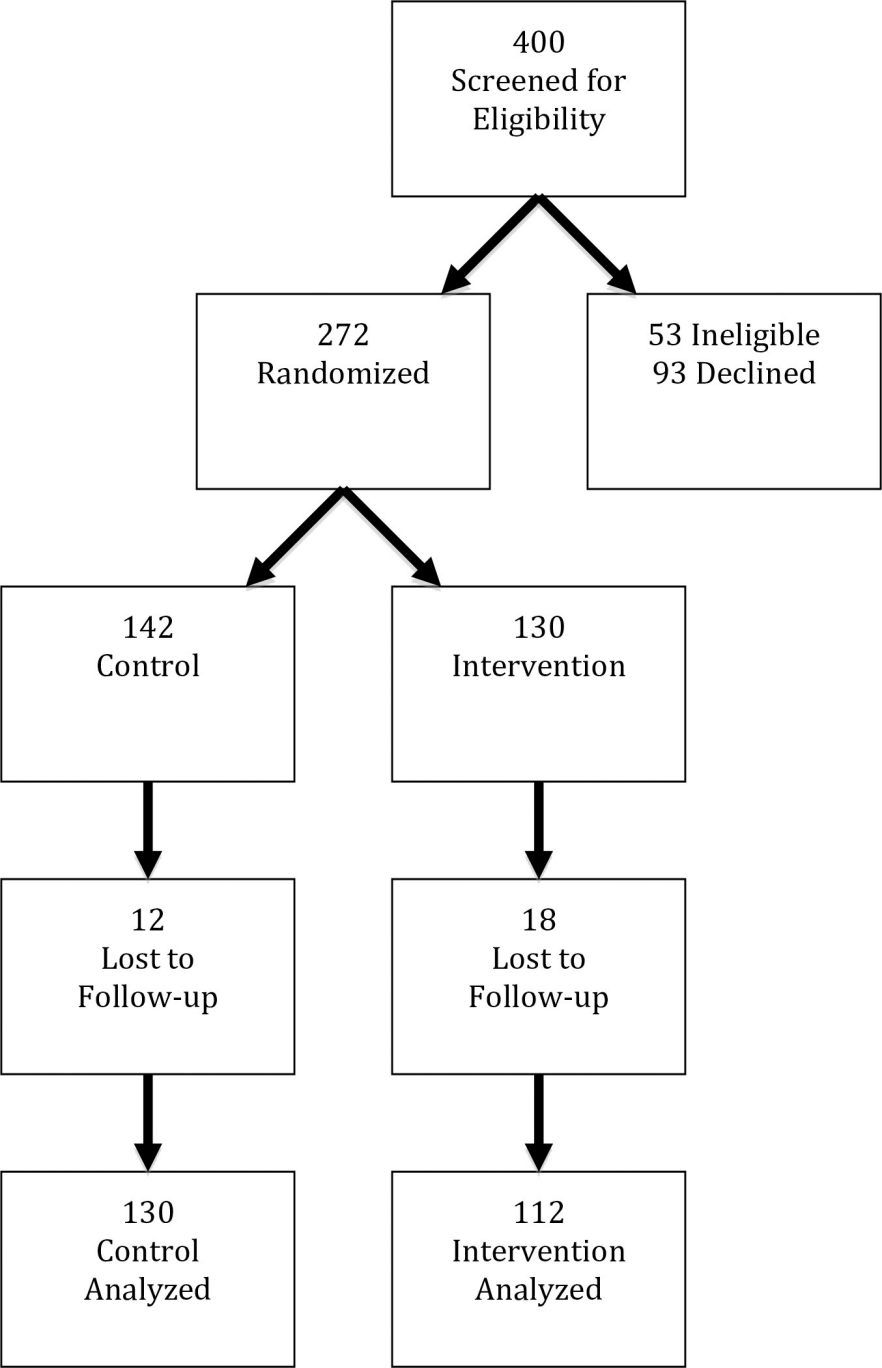
Flow diagram of phases of enrollment, allocation, follow-up and analysis.

Of the 400 participants assessed for eligibility, 53 did not meet eligibility requirements, 93 declined to participate. Two hundred and seventy-two participants were randomized with 142 in the control group and 130 in the intervention group. By 3-month follow-up, 12 in the control group and 18 in the intervention group were lost to follow-up leaving 242 participants (130 control and 112 intervention) included in the analysis (Fig 1) and 242 (89%) of 272 participants completed all study follow-up. There were no differences between the 30 participants lost to follow-up and those that completed the study in baseline demographic data or sexual behaviors nor were there differences between allocation groups in those lost to follow-up. There was very little missing data for baseline demographics. For the race/ethnicity variable there were 2 participants with missing data and for highest education level and health insurance variables there was one participant with missing data. Among the 242 participants who completed follow-up, there was no missing data for variables in the follow-up survey. There were no adverse events in either the control or the intervention group.

Recruitment occurred over approximately 12-months from February 2012 through February 2013 with the 3-month follow-up visits extending to June 2013. Follow-up visits occurred from 2.5 to 4 months after the baseline visit. Participants in the two allocation arms did not differ by demographic characteristics (Table 1). The average age of participants was 21 years and 64.7% of participants were female. The sample represented diverse race/ethnicity with 37.4% self-identifying as white; 34.1% black; 10.0% Asian/Pacific Islander; 7.0% Hispanic; 2.2% Native American; and 9.3% other. The majority reported either no health insurance (57.6%) or Medicaid (12.9%) insurance. Of those 19–24 years of age, only 78.4% had graduated from high school; 59.2% reported at least some college education; and 41.1% were out of school but unemployed (Table 1).

**Table 1:** Demographic characteristics of study participants by allocation arm.

	Control	Intervention	Total
	N = 142	N = 130	N = 272
**Age** mean (range)	21 (15–24)	21(16–24)	21 (15–24)
**Female** n (%)	89 (62.7)	87 (66.9)	176 (64.7)
Male	53 (37.3)	43 (33.1)	96 (35.3)
**Race/Ethnicity**^**a**^ n(%)			
White	56 (39.7)	45 (34.9)	101 (37.4)
Black	47 (33.3)	45 (34.9)	92 (34.1)
Asian/PI	15 (10.6)	12 (9.3)	27 (10.0)
Hispanic	8 (5.7)	11 (8.5)	19 (7.0)
Native American	4 (2.8)	2 (1.6)	6 (2.2)
Other	11 (7.8)	14 (10.9)	25 (9.3)
**Highest Education Level**^**a**^ n (%)			
**Ages 14–18 years**			
< = Some high school	16 (80.0)	8 (72.7)	24 (77.4)
High school graduate	2 (10.0)	3 (27.3)	5 (16.1)
Some college	2 (10.0)	0 (0.0)	2 (6.5)
**Ages 19–24 years**			
< = Some high school	30 (24.6)	22 (18.6)	52 (21.7)
High school graduate	23 (18.9)	23 (19.5)	46 (19.2)
Some college	50 (41.0)	59 (50.0)	109 (45.4)
College graduate	16 (13.1)	12 (10.2)	28 (11.7)
> = Some graduate school	3 (2.5)	2 (1.7)	5 (2.1)
**Employment Status** n (%)			
**Ages 19–24 years**			
Full-time	13 (10.7)	19 (16.0)	32 (13.3)
Part-time	25 (20.5)	29 (24.4)	54 (22.4)
Yes, and in school	10 (8.2)	12 (10.1)	22 (9.1)
No, because in school	15 (12.3)	19 (16.0)	34 (14.1)
No, but looking for work	54 (44.3)	37 (31.1)	91 (37.8)
No, not looking for work	5 (4.1)	3 (2.5)	8 (3.3)
**Health Insurance**^**a**^ n (%)			
Private	18 (12.7)	14 (10.9)	32 (11.8)
Medicaid	19 (13.4)	16 (12.4)	35 (12.9)
None	82 (57.7)	74 (57.4)	156 (57.6)
I don’t know	20 (14.1)	21 (16.3)	41 (15.1)
Other	3 (2.1)	4 (3.1)	7 (2.6)

Chi square and t-tests p<0.05.

^a^Race/ethnicity variable 2 participants with missing data n = 270; highest education level and health insurance variables 1 participant with missing data n = 271.

Participant responses in the control and intervention groups were similar for age of first vaginal sex; number of sex partners; condom and birth control use (e.g. pills, patch, injection, ring, IUD, implant); types of sexual activity; same-sex partner experience; partner concurrency; and STI testing with most recent partner (Table 2). A large proportion of the both the control and intervention groups reported prior pregnancies for self or partner (40.1%). The baseline prevalence of infection from urine NAAT testing was 11.8% for chlamydial infection and 2.6% for gonorrhea. Allocation arms differed in two areas of baseline sexual behavior, with the control group more often reporting a history of sexually transmitted infection (59.2% vs 46.9%, p = 0.04) and ever engaging in transactional sex (9.9% vs 5.4%, p = 0.16) as compared to the intervention group. To account for these differences, these two variables were included in adjusted outcome models. At the end of the baseline visit, 75% of the intervention group reported the interactive computer-based intervention was Very or Extremely Helpful.

**Table 2:** Baseline sexual behaviors of participants by allocation arm.

	Control	Intervention	Total
	N = 142	N = 130	N = 272
**First vaginal sex**			
Age in years			
median^a^ (range)	15.0 (9–22)	16.0 (9–23)	15.0 (9–23)
mean (s.d.)	21.0 (2.16)	21.2 (1.9)	21.1 (2.1)
Condom use n (%)			
Yes	92 (64.8)	95 (73.1)	187 (68.8)
No	50 (35.2)	35 (26.9)	85 (31.2)
Birth control use n (%)			
Yes	19 (13.4)	29 (22.3)	48 (17.7)
No	109 (76.7)	86 (66.2)	195 (71.7)
I don’t know	14 (9.9)	15 (11.5)	29 (10.7)
**Number of vaginal sex partners**			
median (range)			
Last 12 months	3 (1–120)	3 (1–15)	3 (1–120)
Last 2 months	1 (1–25)	1 (1–15)	1 (1–25)
New partners last 2 months	1 (0–15)	1 (0–12)	1 (0–15)
**Unprotected vaginal sex last**			
**2 months** counts of events			
median (range)			
No condom use	4 (0–100)	3 (0–75)	3 (0–100)
No birth control use (females only)	4 (0–80)	5 (0–75)	5 (0–80)
**Other types sex last 2 months**			
n (%)			
Any given oral sex			
Yes	102 (71.8)	102 (78.5)	204 (75.0)
No	40 (28.2)	28 (21.5)	68 (25.0)
Any receptive oral sex			
Yes	113 (79.6)	107 (82.3)	220 (80.9)
No	29 (20.4)	23 (17.7)	52 (19.1)
Any anal sex (given or received)			
Yes	21 (14.8)	19 (14.6)	40 (14.7)
No	121 (85.2)	111 (85.4)	232 (85.3)
**Most recent vaginal sex**			
Condom use n (%)			
Yes	49 (34.5)	50 (38.5)	99 (36.4)
No	93 (65.5)	80 (61.5)	173 (63.6)
Birth control use n (%)			
Yes	55 (38.7)	37 (28.5)	92 (33.8)
No	73 (51.4)	81 (62.3)	154 (56.6)
I don’t know	14 (9.9)	12 (9.2)	26 (9.6)
Tested for STIs prior to sex n (%)			
Yes	50 (35.2)	37 (28.5)	87 (32.0)
No	92 (64.8)	93 (71.5)	185 (68.0)
**Partner has other sex partners**			
n (%)			
Yes	34 (23.9)	26 (20.0)	60 (22.1)
No	55 (38.7)	43 (33.1)	98 (36.0)
I don’t know	53 (37.3)	61 (46.9)	114 (41.9)
**Any same-sex partner last**			
**12 months** n (%)			
Yes	14 (9.9)	17 (13.1)	31 (11.4)
No	128 (90.1)	113 (86.9)	241 (88.6)
**Ever sex while drunk or high**			
n (%)			
Yes	120 (84.5)	107 (82.3)	227 (83.5)
No	22 (15.5)	23 (17.7)	45 (16.5)
**Ever exchange drugs/money for**			
**sex**^**a**^ n (%)			
Yes	14 (9.9)	7 (5.4)^b^	21 (7.8)
No	127 (90.1)	123 (94.6)	250 (92.2)
**Ever pregnant: self or partner**			
n (%)			
Yes	59 (41.6)	50 (38.5)	109 (40.1)
No	83 (58.4)	80 (61.5)	163 (59.9)
**Ever diagnosed with STI** n (%)			
Yes	84 (59.2)	61 (46.9)^b^	145 (53.3)
No	58 (40.8)	69 (53.1)	127 (46.7)
**Baseline urine test results** n (%)			
Chlamydia trachomatis			
Positive	17 (2.0)	15 (11.5)	32 (11.8)
Negative	125 (88.0)	115 (88.5)	240 (88.2)
Neisseria gonorrhoeae			
Positive	3 (2.1)	4 (3.1)	7 (2.6)
Negative	139 (97.9)	126 (96.6)	265 (97.4)

^a^Age first vaginal sex variable two participants excluded for reported age < = 5 years n = 270; Ever exchange drugs/money for sex one participant missing n = 271.

^b^Chi-square and t-tests p<0.05; all other variables were not statistically different between the 2 groups with p>0.05.

### Outcomes comparing intervention and control groups

While these findings did not reach statistical significance, at 3-month follow-up the intervention group reported a 33% lower rate of unprotected vaginal sex (no condom use) [unadjusted IRR = 0.67, 95% CI: 0.44–1.02 p = 0.05] and 20% fewer sex partners [unadjusted IRR = 0.71, 95% CI: 0.50–1.03, p = 0.07] when compared to the control group. As a conservative measure, we adjusted for the baseline differences of self-reported history of STI and ever-transactional sex, which did not change the results (Table 3). Incident STI infection included self-report from interim sexual history via CASI plus results of urine NAAT testing at the 3-month follow-up. There were no gonorrhea cases by self-report or urine testing. Although a rare outcome, there were 13 Chlamydia infections, by self-report and urine testing at follow-up, among 112 participants in the intervention group versus 26 Chlamydia infections among 130 in the control group and was not statistically significant [unadjusted IRR = 0.52, 95% CI: 0.25–1.08, p = 0.08]. Intervention group females reported a lower rate of unprotected vaginal sex (no birth control) than did control group females and was not statistically significant [unadjusted IRR = 0.80, 95% CI: 0.47–1.35, p = 0.40]. Incident pregnancy (self-report and follow-up testing) was also a rare outcome and did not reach statistical significance. There were 50% fewer unintended pregnancies in intervention group females (n = 5) as compared to control group females (n = 10) [unadjusted IRR = 0.51, 95% CI: 0.17–1.58, p = 0.25] (Table 3).

**Table 3:** Primary and secondary analyses comparing intervention to control group: Unadjusted and adjusted outcome models incident rate ratios (IRR) and 95% Confidence Intervals (CI).

Outcomes	Models	Intervention vs Control N = 242
		IRR (95% CI)
Primary		
**Unprotected vaginal sex**^a^	Unadjusted	0.67 (0.44–1.02) p = 0.05
(no condoms)	Adjusted^b^	0.67 (0.44–1.01) p = 0.06
Secondary		
**Number of sex partners**^a^	Unadjusted	0.71 (0.50–1.03) p = 0.07
	Adjusted^b^	0.80 (0.61–1.05) p = 0.11
**Incident CT infection**^c^	Unadjusted	0.52 (0.25–1.08) p = 0.08
(biomarker and self-report)	Adjusted^b^	0.55 (0.26–1.13) p = 0.10
Secondary (females only)		
**Unprotected vaginal sex**^a^	Unadjusted	0.80 (0.47–1.35) p = 0.40
(no birth control)	Adjusted^d^	0.73 (0.42–1.25) p = 0.25
**Incident pregnancy**^c^	Unadjusted	0.51 (0.17–1.58) p = 0.25
(biomarker and self-report)	Adjusted^d^	0.35 (0.10–1.25) p = 0.10

^a^Poisson regression with robust error variance.

^b^Adjusted for baseline differences between intervention and control groups of self-reported history of STI and ever-transactional sex.

^c^Binomial regression.

^d^Adjusted for baseline differences between intervention and control females for self-reported history of ever transactional sex; unprotected sex (no condom); and unprotected sex (no birth control).

In exploratory analyses the models were adjusted for baseline differences between females in the intervention and control groups for self-reported history of ever having transactional sex, unprotected sex (no condom) and unprotected sex (no birth control) in the last 2 months. Intervention females reported fewer partners [adjusted IRR = 0.71, 95% CI 0.50–1.00 p = 0.05] and a significantly lower rate of vaginal sex without condoms [adjusted IRR = 0.50, 95% CI 0.30–0.85 p = 0.01]. In males, there were baseline differences in the intervention and control groups for self-reported history of STIs and unprotected sex (no condom) in the last 2 months. In these adjusted models, no differences were found between intervention and control males for the outcomes of condom use, number of partners or incident STI (Table 4).

**Table 4:** Subset analysis comparing intervention to control females and intervention to control males: Unadjusted and adjusted outcome models with incident rate ratios (IRR) and 95% Confidence Intervals (CI).

Outcomes	Models	Females n = 157	Males n = 85
		Intervention vs Control	Intervention vs Control
		IRR (95% CI)	IRR (95% CI)
**Unprotected vaginal**	Unadjusted	0.67 (0.39–1.18) p = 1.17	0.69 (0.37–1.23) p = 0.24
**sex**^a^ (no condom)	Adjusted	0.50 (0.30–0.85) p = 0.01^b^	0.76 (0.41–1.42) p = 0.39^c^
**Number of sex**	Unadjusted	0.61 (0.37–1.00) p = 0.05	0.98 (0.71–1.35) p = 0.89
**partners**^a^	Adjusted	0.71 (0.50–1.00) p = 0.05^b^	1.08 (0.77–1.52) p = 0.65 ^c^
**Incident CT infection**^d^	Unadjusted	0.80 (0.33–1.96) p = 0.63	0.24 (0.06–0.94) p = 0.04
	Adjusted	0.86 (0.34–2.13) p = 0.74 ^b^	0.31 (0.74–1.32) p = 1.11 ^c^

^a^Poisson regression with robust error variance

^b^ Adjusted for baseline differences between intervention and control females for self-reported history of ever transactional sex; unprotected sex (no condom); and unprotected sex (no birth control).

^c^ Adjusted for baseline differences between intervention and control males for self-reported history of STI; age of first sex; and unprotected sex (no condom).

^d^Binomial regression.

## Discussion

In this pilot randomized controlled trial, execution of the interactive computer-based intervention proved feasible in a clinic setting and acceptable to participants. At 3-month follow-up, there were non-significant reductions in unprotected vaginal sex; number of sex partners; incident STI; and unintended pregnancy. However, in an exploratory subset analysis of all females, there was a statistically significant reduction in vaginal sex without condoms.

The procedures of the baseline study visit for the intervention and control groups were identical, except that the intervention group remained on the computer for 15–20 minutes longer than the control group to complete the intervention. This study did not use a placebo intervention for the control group to match the additional time that the intervention group was on the computer. The time spent by each participant with a clinician during the clinic visit was per clinic standard and not prescribed by the research study, and did not differ by allocation arm.

Research staff and participants were blinded as to allocation group. As randomization, allocation to treatment arm, and engagement in the intervention all occurred on the computer with the participant alone in a private exam room, there was little risk for blinding failure. Participants did not know what content to expect in the intervention, as during the consent process they were only informed that researchers were testing different ways to discuss sexual health with young people. Because the intervention group spent more time on the computer than the control group it is possible that the research assistant, who was guiding the participant through the study visit, could surmise allocation group by the end of the visit; however, the interaction with the research assistant after the computer portion of the visit was designed to avoid impact on outcomes. Five written questions about content of counseling messages were asked of participants who had clinician-visits; followed by logistics of incentive payment and study follow-up appointment.

The intervention was delivered via computer program to assure standard delivery to all participants. Although STI clinicians were not part of the intervention, participants received standard of care during their clinic visit, which for some included an encounter with a clinician. The expertise and predilection for providing care to adolescents and young adults differs amongst STI clinicians. While standard of care for the clinic includes risk reduction counseling with the clinician, it is possible that the quality of these discussions varied amongst participants; however, there is no reason to believe these differences were unevenly dispersed between allocation arms, and clinicians were not aware of whether or not their patients received the study intervention.

To strengthen the generalizability of this interactive computer-based intervention, it delivered standard content on a sensitive health topic with fidelity that does not depend on the expertise, biases, or priorities of any individual clinician.

Comparator and treatment arms received standard of care from the STI Clinic. As all participants may have benefited from the risk-reduction counseling of an STI clinician, it is possible that if this intervention was tested in a general medical clinic, with providers that do not specialize in sexual health, the impact of the intervention may have demonstrated a greater difference between intervention and control groups.

While higher risk patients, like those enrolled in this study, may benefit most from a risk-reduction intervention, this ICBI contains basic safer-sex, STI prevention and contraception information and skill building which is important for all adolescents and young adults. The study sample that tested our intervention spanned a decade of life marked by significant physical growth, psychosocial development and cognitive maturity and included both males and females. The results of the exploratory analysis indicate the intervention may have been more effective in females than in males. Tailoring the intervention content to specific populations may strengthen its impact.

We addressed the limited and sporadic healthcare-seeking behavior and low rates of keeping return appointments in adolescents and young adults, by designing the ICBI as a one-session intervention administered during one clinic visit. The time it took to complete the intervention, in the context of a clinic visit, could easily be achieved in a waiting room or exam room. Costs are contained with this computerized intervention, housed on a server and deliverable to any clinic or location with Internet access.

Our findings support other results reported in the literature that have found ICBIs are acceptable and feasible, and potentially effective in reducing sexual risk behaviors. While underpowered to detect statistical significance, our findings are unique in showing consistent reductions in unprotected sex and number of sex partners at 3-month follow-up after only one brief session during one clinic visit. Similarly, our study adds promising reductions in STI and unintended pregnancy outcomes.

The computer intervention tested in this study stands alone from others in the literature in that it is delivered as a one-session intervention during one clinic visit in the context of adolescent and young adult patients seeking care. The design of the intervention is unique as it utilizes physician avatars to mimic a provider-patient interaction; we found no other such intervention yet published for adolescents and young adults. One study of HIV-positive substance-abusing middle-aged adults, used actor-portrayed video doctors to provide tailored feedback, with a booster session at 3 months, and found reduced illicit drug use and unprotected sex in the intervention group compared to the control group [48].

ICBIs are considered interactive because participants actively respond to the computer program; but not all such interventions offer feedback tailored to an individual’s specific risk behaviors. Of the four randomized controlled trials that provided risk-reduction feedback individualized to adolescents and young adults [28, 29, 39, 49], only one trial included participants less than 18 years old; recruited from a clinic population; and tested biomarkers [28]. Two studies had upper age ranges exceeding 25 years and were in high-risk populations such as those with same-sex partners, substance abuser, sex workers, or individual previously incarcerated [39, 49]. Our intervention gives personalized, confidential feedback to each individual based upon sexual risk history, motivation, and perceived barriers to behavior change. The intervention is self-paced and enables the participant to choose which sexual health topics to explore. It is innovative in that it asks the participants to identify and commit to a follow-up behavioral change goal of their choosing, which is a hallmark of motivational interviewing. Only one other computer-based intervention utilized this strategy; it was tested in a population of psychology class college students, who reported increased condom use at 4 weeks follow-up[29].

Prevention and risk-reduction research in adolescents and young adults has been historically segregated along the lines of the target behaviors, with outcomes including either condom use and STI/HIV *or* birth control use and unintended pregnancy prevention. The present study is unique in addressing both STI/HIV and unintended pregnancy as co-morbid outcomes of risky sexual behavior. Most previous studies measured knowledge, attitude, and self-efficacy outcomes, with many also measuring behavioral outcomes, such as condom use or number of sex partners [27–32, 38–40, 49–51], but only three studies collected biomarker outcomes for STI or pregnancy [27, 40, 52].

This study was limited by a relatively short follow-up interval of 3-months and does not provide information on sustainability of the intervention impact. Lower than expected clinic patient volumes resulted in a lower than expected number of study participants. However, for those enrolled, the study had a high retention rate. Although underpowered to reach statistical significance, we did observe a consistent and substantial decline in risk behaviors and biomarker outcomes.

Future studies need to engage larger samples to determine effectiveness of the ICBI for reducing unprotected sex, STI infection and unintended pregnancy with longer follow-up needed to understand sustainability of intervention impact. This intervention creates an opportunity to provide and receive confidential sexual health information in a private setting; it is well suited to tailor for and test in special populations including adolescent and young adult females; transgender individuals; and males who have sex with males.

## Supporting information

S1 Checklist CONSORT checklist. (DOC)

S1 Protocol Study Protocol Document 1. (PDF)

S2 Protocol Study Protocol Document 2. (DOC)
