# Supplementary material for: Results from e-KISS: electronic-KIOSK Intervention for Safer Sex: A pilot randomized controlled trial of an interactive computer-based intervention for sexual health in adolescents and young adults
Source: PLoS One. 2019 Jan 23;14(1):e0209064. doi: 10.1371/journal.pone.0209064 (PMC6343886; doi:10.1371/journal.pone.0209064)
Supplement: S1 Protocol — (PDF) [file pone.0209064.s002.pdf]

RECEIVED
Human Subjects D[illegible]

DEC 15 20[illegible]

UW

# Study Protocol Steps, Randomization and Oral Script for Research Assistant

**Patient registration and sexual history per clinic routine**
Patient registers at front desk in STD Clinic per clinic routine process.
They are directed to STD Clinic computer and enter their sexual history in STD KIOSK per clinic routine standard.

**FIRST screen for eligibility by use of STD Computer**
The First screen for eligibility for the study is done with the information generated by the STD registration computer KIOSK as per clinic standard; each chart contains a computer generated triage form containing information about their age and sexual history. The RA will scan the triage sheets for 2 eligibility criteria: age 14-24 years and vaginal sex in the last 2 months. The RA will approach potential participants in the waiting room by indentifying them with their clinic number pull. The RA will tell the potential participant that they might be eligible for a research study and ask them if they would like to hear more. If they would like to hear more, the RA will escort them to a private clinic room to provide more information about the study. If they say no, the RA will thank them for their time and will not have further contact with the patient.

**If they would like to hear more**, the RA will escort the potential participant to a private clinic room to provide more information about the study and answer all questions. The RA will say; "Hi, I am "XX". The University of Washington, with the STD Clinic, is doing a research study in the STD Clinic to learn more about the sexual health of teenagers and young adults. We are trying to learn ways to discuss safe-sex with young people using a computer. You may be eligible to participate. The study will take less than an hour to do and we will compensate you for your time. Being in the study or deciding not to be in the study will not change the care you receive in clinic today or in the future."

**SECOND screen for eligibility by UW study computer**
"First we need to see if you are eligible for the study. To do that we need you to answer a few questions on the computer."
RA logs patient onto study computer and says they are available for questions at any time.
Patient will be identified on computer by a participant ID number assigned to them by the computer program.
RA steps out of room while patient answers screening questions.
"I will return when you are finished with the screening questions. Please crack the door open so that I know you are ready."
Patient answers additional screening questions on study computer.
RA confirms eligibility on the computer. They are then provided Information Sheet (consent/assent) on computer.
RA will take a urine sample from the females to test for pregnancy. If the female is pregnant, she is no longer eligible for the study (The participant is informed of this via



APPROVED
DEC 19 2011
UW Human Subjects
Review Committee

Information Statement on the computer). If the potential participant is pregnant, she will return to the waiting room and her visit will go as standard clinical procedure. RA will reiterate the importance of the return 3 month follow up appointment.

**Those screened but not in the study**
If NOT eligible:
"I am sorry you are not eligible for the study or this study is not right for you. Thank you for your time. I will take you back to the waiting room and you will go on with your clinic visit as usual"
RA escorts them back to clinic waiting room and they resume their visit per clinic routine.

**If eligible but decide NOT to participate:**
"That's fine. Thank you for your time. I will take you back to the waiting room and you will go on with your clinic visit as usual."
RA escorts them back to clinic waiting room and they resume their visit per clinic routine.

**Those eligible and want to participate**
RA available to answer questions.
After agree to be in study they enter their name and contact information.
RA confirms entered all data.
RA fills out on the contact information that the participant gave on the computer. The RA verifies it is the same information and valid. RA goes over with the participant how frequent they will be contacted and under which circumstances the RA would call the contact provided by the participant.

**HIPAA Authorization form signed here.**

**Those eligible but do not enter all contact information**
If they don't enter all contact information they are prompted by the computer that they need to do this in order to participate. If they want to withdraw then they notify RA. "That's fine. Thank you for your time. I will take you back to the waiting room and you will go on with your clinic visit as usual."
RA escorts them back to clinic waiting room and they resume their visit per clinic routine. RA has not further contact with them.

**Randomization to control vs intervention group**
At this point participant is randomized into control vs intervention group by the computer.
It will be a stratified randomization by gender, age group, and type of clinic visit.
Both control and intervention groups next answer additional sexual history questions.
Control group stops computer program after enters sexual history questions.
Intervention groups stops computer program after the intervention.



APPROVED
DEC 19 2011
UW Human Subjects
Review Committee

For both groups after completion of the computer program check in with RA. RA answers any questions.

**Verification of contact information, follow-up method**
RA verifies contact info entered into computer and best way to follow-up with them in 3 months.

**Explanation of 3 month follow-up visit**
RA explains follow-up visit in 3 months.

"We need you to come back in 3 months for a study follow-up visit with me or one of the other RAs. When you return we will ask you to answer a computer survey about your sexual health. Then, as part of the study, we will ask you to provide a urine sample to test for gonorrhea and Chlamydia (or females may provide a self-obtained vaginal swab). Females will be asked to provide a urine sample for pregnancy testing. We expect that it will take less than an hour for you to complete the study follow-up visit. When you return we will ask you to meet with the research assistant only. You are not required to have a regular clinic visit unless you want to.

If you complete the follow-up visit you will receive $50 as a token of our appreciation and a bus ticket home if you need one. I will contact you in about 2 months to set up an appointment. You will not have to wait to be seen as you will have a specific time set up to see me."

**Next steps for today's visit**
RA tells them what to expect next today.
"Now I will take you back to the waiting room and you will go on with your clinic visit as usual. As part of your clinic visit today you will be tested for gonorrhea and Chlamydia which can be done with a urine sample or genital swab (talk with your clinician about the best test for you). Females will be asked to provide a urine sample for pregnancy testing. When you are finished seeing the clinician come back here and you will receive $25 as a token of our appreciation and a bus ticket home if you need one."

**Debriefing and Exit Interview**
When the participant returns to the RA to receive the $25 they will be debriefed about the intervention.
Debrief about Intervention:
"As you know we are trying to learn ways to discuss safer-sex with young people and to do that in this study half of the participants received the computer intervention and half did not. It is completely random who gets it and who doesn't; a computer program determines that, so I don't even know if you received the intervention today or not. I am sorry that I was unable to tell you this before you started the study today but I didn't because when some people know they are getting the intervention it changes how they



APPROVED
DEC 19 2011
UW Human Subjects
Review Committee

answer the questions and I didn't want that to happen to you. Do you have any questions about that?"

Exit Interview for those participants that have a clinician visit.

RA will then proceed with the Exit Interview

RA will then set up a three month appointment at this time and hand the appointment card to the participant.



APPROVED
DEC 19 2011
UW Human Subjects
Review Committee
