# Supplementary material for: Results from e-KISS: electronic-KIOSK Intervention for Safer Sex: A pilot randomized controlled trial of an interactive computer-based intervention for sexual health in adolescents and young adults
Source: PLoS One. 2019 Jan 23;14(1):e0209064. doi: 10.1371/journal.pone.0209064 (PMC6343886; doi:10.1371/journal.pone.0209064)
Supplement: S2 Protocol — (DOC) [file pone.0209064.s003.doc]

**Study Protocal Steps, Randomization and Oral Script for Research Assistant**

**Patient registration and sexual history per clinic routine**

Patient registers at front desk in STD Clinic per clinic routine process.

They are directed to STD Clinic computer and enter their sexual history in STD KIOSK per clinic routine standard.

**FIRST screen for eligibility by use of STD Computer**

The First screen for eligibility for the study is done with the information generated by the STD registration computer KIOSK as per clinic standard; each chart contains a computer generated triage form containing information about their age and sexual history. The RA will scan the triage sheets for 2 eligibility criteria: age 14-24 years and vaginal sex in the last 2 months. The RA will approach potential participants in the waiting room by identifying them with their clinic number pull. The RA will tell the potential participant that they might be eligible for a research study and ask them if they would like to hear more. If they would like to hear more, the RA will escort them to a private clinic room to provide more information about the study. If they say no, the RA will thank them for their time and will not have further contact with the patient.

**If they would like to hear more,** the RA will escort the potential participant to a private clinic room to provide more information about the study and to answer all questions. The RA will say, “Hi, I am ‘XX’. The University of Washington, with the STD Clinic, is doing a research study in the STD Clinic to learn more about the sexual health of teenagers and young adults. We are trying to learn ways to discuss safe-sex with young people using a computer. You may be eligible to participate. The study will take less than an hour to do and we will compensate you for your time. Being in the study or deciding not to be in the study will not change the care you receive in the clinic today or in the future.”

**SECOND screen for eligibility by UW study computer**

“First we need to see if you are eligible for the study. To do that we need you to answer a few questions on the computer.”

RA longs patient onto study computer and says they are available for questions at any time.

Patient will be identified on computer by a participant ID number assigned to them by the computer program.

RA steps out of room while patient answers screening questions.

“I will return when you are finished with the screening questions. Please crack the door open so that I know you are ready.”

Patient answers additional screening questions on the computer. They are then provided Information Sheet (consent/assent) on computer.

RA will take a urine sample from the females to test for pregnancy. If the female is pregnant, she is no longer eligible for the study (The participant is informed of this via Information Statement on the computer). If the potential participant is pregnant, she will return to the waiting room and her visit will go as standard clinical procedure. RA will reiterate the importance of the return 3 month follow up appointment.

**Those screened but not in the study**

If NOT eligible:

“I am sorry you are not eligible for the study or this study is not right for you. Thank you for your time. I will take you back to the waiting room and you will og on with your clinic visit as usual.”

RA escorts them back to clinic waiting room and they resume their visit per clinic routine.

**If eligible but decide NOT to participate**

“That’s fine. Thank you for your time. I will take you back to the waiting room and you will go on with you clinic visit as usual.”

RA escorts them back to clinic waiting room and they resume their visit per clinic routine.

**Those eligible and want to participate**

RA available to answer questions.

After agree to be in study they enter their name and contact information.

RA confirms entered all data.

RA fills out the contact information that the participant gave on the computer. The RA verifies it is the same information and valid. RA goes over with the participant how frequent they will be contacted and under which circumstances the RA would call the contact provided by the participant.

**HIPAA Authorization form signed here.**

**Those eligible but do not enter all contact information**

If they don’t enter all contact information they are prompted by the computer that they need to do this in order to participate. If they want to withdraw then they notify RA. “That’s fine. Thank you for your time. I will take you back to the waiting room and you will go on with your clinic visit as usual.”

RA escorts them back to clinic waiting room and they resume their visit per clinic routine. RA has not further contact with them.

**Randomization to control vs intervention group**

At this point participant is randomized into control vs intervention group by the computer.

It will be stratified randomization by gender, age group, and type of clinic visit.

Both control and intervention groups next answer additional sexual history questions. Control group stops computer program after sexual history questions.

Intervention group stops computer program after the intervention.

For both groups after completion of the computer program check in with RA.

RA answers any questions.

**Verification of contact information, follow-up method**

RA verifies contact info entered into computer and best way to follow-up with them in 3 months.

**Explanation of 3 month follow-up visit**

RA explains follow-up visit in 3 months.

“We need you to come back in 3 months for a study follow-up visit with me or one of the other RAs. When you return we will ask you to answer a computer survey about your sexual health. Then as part of the study, we will ask you to provide a urine sample to test for gonorrhea and Chlamydia (or females may provide a self-obtained vaginal swab). Females will be asked to provide a urine sample for pregnancy testing. We expect that it will take less than an hour for you to complete the study follow-up visit. When you return we will ask you to meet with the research assistant only. You are not required to have a regular clinic visit unless you want to.

If you complete the follow-up visit you will receive $50 as a token of our appreciation and a bus ticket home if you need one. I will contact you in 6 weeks as a reminder of your follow up date and time. You will not have to wait to be seen as you will have a specific time set up to see me. I will also call you 24 hours before your scheduled follow up visit to remind you of your appointment.”

**Next steps for today’s visit**

RA tells them what to expect next today.

“Now I will take you back to the waiting room and you will go on with your clinic visit as usual. As part of your clinic visit today you will be tested for gonorrhea and Chlamydia which can be done with a urine sample or genital swab (talk with you clinician about the best test for you). When you are finished seeing the clinician come back here and you will receive $25 as a token of our appreciation and a bus ticket home if you need one.”

**Debriefing and Exit Interview**

When the participant returns to the RA to receive the $25 they will be debriefed about the intervention.

Debrief about intervention:

“As you know we are trying to learn ways to discuss safer-sex with young people and to do that in this study half of the participants received the computer intervention and half did not. It is completely random who gets it and who doesn’t; a computer program determines that, so I don’t even know if you received the intervention today or not. I am sorry that I was unable to tell you this before you started the study today but I didn’t because when some people know they are getting the intervention it changes how they answer the questions and I didn’t want that to happen to you. Do you have any questions about that?”

Exit interview for those participants that have a clinician visit.

RA will then proceed with the Exit Interview.

RA will then set up a three month appointment at this time and hand the appointment card to the participant.

**e-KISS Retention Protocol**

**Goal:** To retain >90% of study participants for their 3 month follow up visit for e-KISS.

**Method:**

All participants at time of screening

1. The recruiter will discuss with all potential participants the importance of the one time 3 month follow up visit to the outcome of the study.
2. Confirm with participants their ability to come to the scheduled 3 month follow up visit as they are described.
3. Explain to the participant how it is worse for the study for a participant to enroll and drop out later, than not to have enrolled at all. Once participants have committed to the study we cannot replace them with somebody else.
4. Reiterate why retention is important in this study. We have to have enough people in order to have reliable data. If we do not have a certain number of participants who finish the study then we might end up with results that don’t clarify whether the counseling method is effective and worth implementing for the public.

After the participant has consented to being in the study

1. We will collect from the participant: current address, home/cell telephone numbers, email address and the first name and telephone number of a contact person to call in case we are unable to reach the participant.
2. It is very important that the participant call us in advance if they cannot attend the scheduled follow up appointment.
3. The participant will be given an appointment card with the date, time and address for the follow up visit. On the appointment card is also the contact information for the research assistant (RA).
4. The RA will contact the participant 6 weeks after their initial visit for the study and then again 1 week prior to the follow up visit. Contact will also be made 24 hours in advance of the appointment.
5. If the RA has not heard from the participant to confirm the appointment after the 1 week reminder contact has been made, the RA will call the contact in effort to get a response from the participant.
6. If a participant does not show up for the scheduled appointment the RA will contact them 30 minutes after their scheduled appointment. A total of 10 attempts over three weeks; phone, email, and/or letter, will be made to make contact with the participant to reschedule. During these attempts, the RA will ask the participant if they are interested in continuing participation. If no response is given, the RA will consider this person lost to follow up.

If a participant is unable to return to the study site to complete the 3 month follow-up

Some participants may move away from Seattle or be otherwise unable to return to the study site for the 3 month follow-up visit. These individuals will be given the option of completing none, one, or both of the following remote follow-up procedures. The RA will discuss these options over the phone with the following script:

“I know you cannot return to the clinic to complete the 3 month follow-up visit with the e-KISS study like we originally planned. We have procedures in place so that you can complete some of the follow-up remotely, without coming in to the clinic. These procedures are completely optional, and you may choose to complete none, one, or both of the optional procedures. Both procedures will require that you provide me with a mailing address where I can send you letters or a small parcel. Would you like to hear about the two optional procedures you may choose to complete?”

If yes, the RA will explain the two remote follow up options:

**Option 1.** Participants may choose to complete the e-KISS 3 month follow-up survey remotely on a personal or public computer. The RA will explain that this is identical to the survey that the participant would have taken at the study site during the follow up visit. The follow-up survey is shorter than the initial survey and involves answering questions similar to the initial e-KISS survey through a web browser connected to the internet. The survey takes about 15 minutes. In addition, the participant must provide a physical address to receive the $25 compensation for completing this remote follow-up option. If the participant wishes to complete this remote survey option, the RA will give the participant instructions for accessing and completing the survey as the participant prefers; over the phone, by email, or by letter. Accessing the survey involves entering the 3 month follow-up survey URL into a web browser and entering the participant’s unique Custom ID number into the survey. If the survey is interrupted and the participant needs to resume it, the same survey URL and Custom ID number can be used to resume the survey. Once the survey is complete, it will automatically register with the DatStat server online. Once the survey is registered as complete, the RA will send a letter thanking the participant for their participation in e-KISS with a check or gift card for $25 enclosed. The letter will not make reference to the University of Washington, the Public Health – Seattle & King County STD Clinic or the general or specific purpose of the study.

**Option 2.** Participants may choose to complete gonorrhea and Chlamydia testing remotely by self-collecting genital swab or urine sample and returning them to the study site by mail. The RA will explain that this will involve receiving a parcel consisting of a standard padded envelope that will contain the specimen collection kit, instructions for collection, and a self-addressed, stamped, small padded envelope for returning the specimen. The participant will self-obtain either a vaginal swab (for females) or a urine sample (for males), routine procedures available to patients in the STD clinic. Participants will be asked to seal the specimen tube, place it within two small “biohazard” bags, and place the return envelope containing the specimen in the mail. These procedures should take 15 minutes or less. The specimen tube will be labeled with the participant’s study ID number only, and no name or identifying information will be present on the specimen tube, return envelope, or anywhere within the return parcel to protect confidentiality. In order to complete this optional follow-up procedure, participants must be willing to provide a mailing address. Once the specimen kit is received at the STD clinic, the RA will contact the participant by phone to discuss how he/she can retrieve the results from the gonorrhea and Chlamydia testing. The RA will also send a letter thanking the participant for their participation in e-KISS with a check or gift card for $25 enclosed. The letter will not make reference to the University of Washington, the Public Health – Seattle & King County STD Clinic or the general or specific purpose of the study.

“Now that I’ve told you about the two remote follow-up options you may complete, do you have any questions about those options or what we would ask you to do?”

RA answers all questions.

“You do not have you complete either of these options. If you do not wish to complete either option, you will be disenrolled from the study and I will not contact you again. You may complete either one of these options on its own. If you decide to complete just one of the follow up options, you will receive $25 compensation in the mail as a token of our thanks once you’re all finished. You may also decide to complete both of the follow up options. If you decide to complete both, you will be mailed $25 after each component is finished, for a total of $50 as a token of our thanks. Do you need some time to think about these options or do you want to make your decision now?”

If participant decides to complete one or both, a current mailing address will be obtained as well as the participant’s preference for receiving instructions by mail or by email for the remote survey if it is chosen.
